# Follow the Path: Unveiling an Azole Resistant *Candida parapsilosis* Outbreak by FTIR Spectroscopy and STR Analysis

**DOI:** 10.3390/jof10110753

**Published:** 2024-10-30

**Authors:** Elena De Carolis, Carlotta Magrì, Giulio Camarlinghi, Vittorio Ivagnes, Bram Spruijtenburg, Eelco F. J. Meijer, Cristiano Scarselli, Eva Maria Parisio, Maurizio Sanguinetti

**Affiliations:** 1 Dipartimento di Scienze di Laboratorio ed Ematologiche, Fondazione Policlinico Universitario “A. Gemelli” IRCCS, 00168 Roma, Italy; carlotta.magri@unicatt.it (C.M.); vittorio.ivagnes@unicatt.it (V.I.); maurizio.sanguinetti@policlinicogemelli.it (M.S.); 2 Microbiology Unit, S. Donato Hospital, 52100 Arezzo, Italy; giulio.camarlinghi@uslsudest.toscana.it; 3 Radboudumc-CWZ Center of Expertise for Mycology, 6532 SZ Nijmegen, The Netherlands; b.spruijtenburg@cwz.nl (B.S.); eelco.meijer@cwz.nl (E.F.J.M.); 4 Canisius-Wilhelmina Hospital (CWZ)/Dicoon, 6532 SZ Nijmegen, The Netherlands; 5 Tuscany Rehabilitation Clinic, 52025 Montevarchi, Italy; scarselli@crtspa.it

**Keywords:** azole resistance, *Candida parapsilosis*, FTIR spectroscopy, nosocomial infections, outbreak management, Short Tandem Repeats

## Abstract

Accurate identification and rapid genotyping of *Candida parapsilosis*, a significant opportunistic pathogen in healthcare settings, is crucial for managing outbreaks, timely intervention, and effective infection control measures. This study includes 24 clinical samples and 2 positive environmental surveillance swabs collected during a fluconazole-resistant *Candida parapsilosis* outbreak at the Tuscany Rehabilitation Clinic (Clinica di Riabilitazione Toscana, CRT), located in the province of Arezzo, Italy. Fourier-transform infrared (FTIR) spectroscopy, genetic sequencing of the ERG11 gene, and short tandem repeat (STR) analysis was applied to track the fluconazole-resistant *C. parapsilosis* outbreak at the CRT facility. FTIR analysis clustered the isolates into two major groups, correlating with resistance-associated *ERG11* mutations (Y132F and R398I), azole resistance levels, and year of isolation. The combined use of FTIR spectroscopy and STR typing provided a comprehensive approach to identify and track fluconazole-resistant *C. parapsilosis* isolates, which identified specific clusters of genetically similar isolates. By comparison with feasible molecular techniques, we conclude that FTIR spectroscopy applied in real time can inform targeted infection control strategies and aid in the effective management of nosocomial infections.

## 1. Introduction

*Candida parapsilosis* is a significant opportunistic pathogen in healthcare settings, particularly affecting immunocompromised individuals. Known for causing a range of infections, from superficial skin conditions to severe systemic diseases such as bloodstream infections (candidemia), it has become a major concern in hospitals and other healthcare facilities. In particular, the last decades experienced an alarming increase in the number of *C. parapsilosis* isolates accounting for 25% of all candidemia cases, along with a significant rise of azole resistance in Asia, Latin America, South Africa, and mainly in Southern Europe [1,2,3,4]. Mutations in the *ERG11* gene, which encodes the enzyme lanosterol α-14-demetylase, are the primary mechanism of fluconazole resistance in *C. parapsilosis* [4,5]. This is a major concern, narrowing of the range of therapeutic options available to combat this emerging pathogen. Clonal *C. parapsilosis* outbreaks have been reported, particularly in intensive care units and among immunocompromised patients, posing significant challenges for infection control and treatment [6]. Due to the species high propensity for patient-to-patient transmissions, and prolonged persistence on inanimate surfaces [7], rapid and accurate identification complemented with genotyping of this pathogen in terms of temporal spread and genomic reconstruction is crucial in managing outbreaks, as it allows for timely intervention and the implementation of effective infection control measures.

Traditional methods of *C. parapsilosis* outbreak detection and tracking, such as culture-based techniques and polymerase chain reaction (PCR), often fall short in terms of speed and resolution. These conventional approaches can be labor intensive, time and cost consuming, and cannot distinguish between closely related isolates, complicating outbreak investigations and delaying appropriate responses [8].

IR-Biotyper, a Fourier-transform infrared (FTIR) spectroscopy-based tool, has recently become available for real-time microbial genotyping [9,10]. FTIR spectroscopy measures the absorption of infrared light by chemical bonds within a molecule, producing a unique spectral fingerprint that can be used for grouping in cluster-related isolates. This technology represents a significant leap forward in the field of clinical microbiology, offering rapid, precise, and high-resolution typing of bacterial pathogens and yeasts. By analyzing the unique infrared spectral fingerprints of microbial samples, the FTIR Biotyper can differentiate between species and even subspecies, providing critical insights into the epidemiology of infections.

A study by Essendoubi et al. (2005) [11] demonstrated that the FTIR Biotyper could identify various *Candida* species often within a matter of minutes. This capability is particularly valuable in outbreak scenarios where timely information is paramount. Unlike traditional methods that may take days to yield results, the FTIR Biotyper can allow for almost real-time monitoring of pathogen spread, enabling quicker response measures.

In this article, we explore the use of the Bruker FTIR Biotyper in tracking a *C. parapsilosis* outbreak and identify the source of infection. The outbreak occurred at the Tuscany rehabilitation clinic (CRT) in Arezzo, Italy. CRT specializes in the treatment of patients with severe acquired brain injuries and those discharged from hospitals with complex disabilities, including neurological, orthopedic, and cardiological conditions, requiring continuous 24 h medical and nursing care. The facility is equipped with double-occupancy hospital rooms and shared gyms accessible to all patients. The rehabilitation center showed a notably higher percentage of *C. parapsilosis* positive blood cultures both in 2022 and 2023 (85% and 73.8%, respectively) compared to the hospital center in which it is located (32.9% and 34.9%, respectively). Moreover, a higher fluconazole resistance rate was observed in the CRT with respect to the hospital, in particular 26.5% vs. 4.2% in 2022 and 44% vs. 18% in the year 2023. Through the examination of FTIR and STR (short tandem repeat) application to this outbreak scenario, we highlight the benefits in enhancing outbreak management and implement control hygiene measures when needed. Considering that existing literature on the item mainly involves slow and costly genotyping methods having no impact for a real-time surveillance strategy and addressing fungal nosocomial clusters as unpredictable [12], the present study aims to propose a valid and feasible alternative to be applied in real time during a suspected nosocomial fungal outbreak.

## 2. Materials and Methods

### 2.1. Isolates Collection

In this study, 24 clinical samples and 42 non-repetitive surface swabs collected in years 2022 and 2023 at the CRT facility (Table S2) were retrieved from patients’ blood cultures and from six patients’ rooms (30 swabs), common rooms as gym, doctors’ rooms, healthcare staff kitchen, and bathroom (12 swabs). The environmental swabs were cultured on Sabouraud agar (Oxoid, UK) and Columbia blood agar (Biomerieux, France). *C. parapsilosis* isolates were identified by MALDI-TOF MS. Specifically, two of these surveillance swabs collected from the monitor (CP-AR-542) and over-bed table (CP-AR-544) turned out to be fluconazole resistant (Table S1) and were included in the analysis.

### 2.2. Antifungal Susceptibility, ERG11 Sequencing, FTIR and STR Analysis

Fluconazole susceptibility testing was performed using ITAMYUCC YeastOne plates (Thermo Fisher Scientific Inc., Waltham, MA, USA), following the manufacturer’s protocol. Minimum inhibitory concentration (MIC) values were determined as the lowest concentration of antifungal drugs inhibiting the visible growth and interpreted according to CLSI guidelines [13].

**Figure 1:**
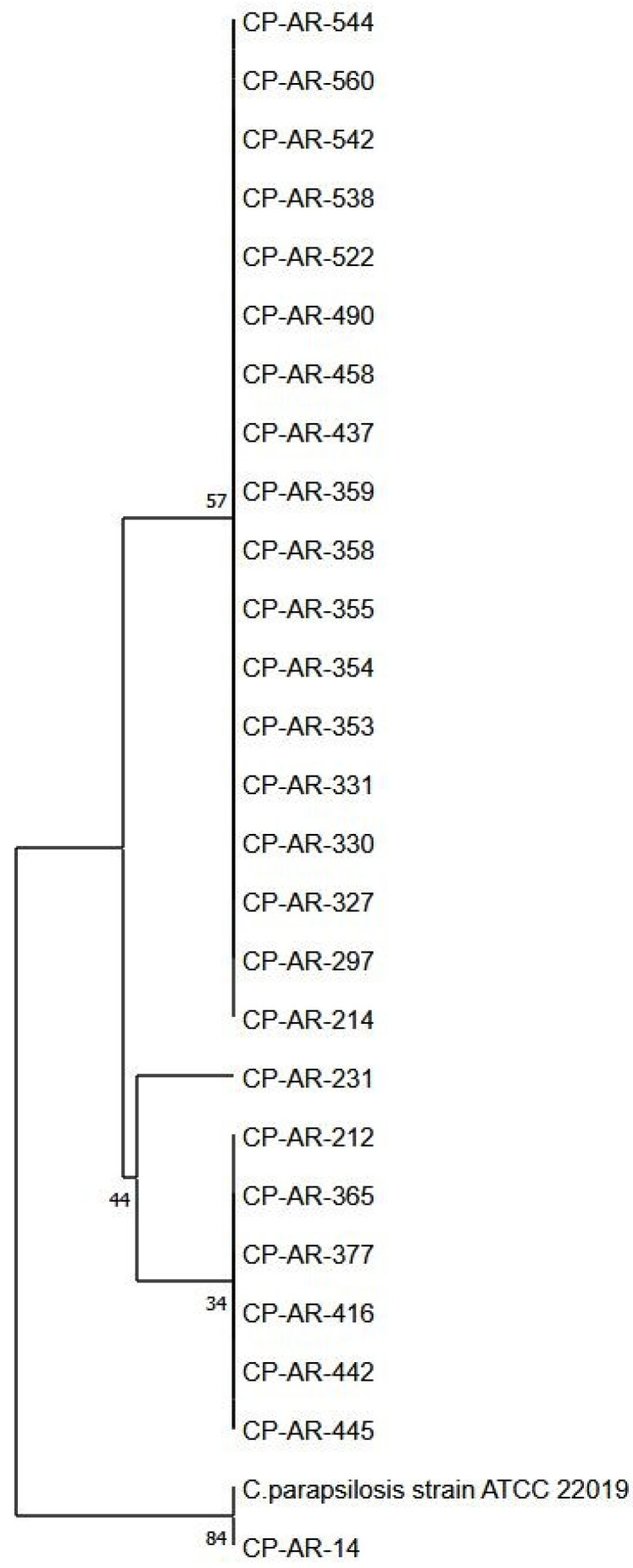
Phylogenetic tree of *ERG11* gene sequences among *Candida parapsilosis* isolates.

Genomic DNA was extracted from each sample using the DNeasy Plant Mini Kit (Qiagen, Germany) and stored at −20 °C until further analysis. *C. parapsilosis* isolates were characterized for the presence or absence of point mutation(s) in the *ERG11* gene. In particular, sequencing was performed using previously described specific primers designed to amplify the *ERG11* gene [14]. The amplified DNA fragments were then sequenced using the SeqStudio Genetic Analyzer (Thermo Fisher Scientific). Point mutations in the ergosterol gene were detected by *ERG11* gene alignment by MEGA11 software v. 11.0.10 [15]. A dendrogram was constructed including all the fluconazole-resistant isolates by unweighted pair group mean average (UPGMA) clustering method, and distances were calculated by the maximum likelihood composite method. Bootstrap test results generated from 100 replicates are included next to the branches. *ERG11 C. parapsilosis* ATCC 22019 gene sequence was included as tree-root (Figure 1).

For FTIR analysis, *C. parapsilosis* isolates from three biological replicates grown on Saboraud dextrose agar plates (Kima, Padua, Italy) for 24 h, following the manufacturer instructions. Briefly, after suspension in 50 µL of 70% (*v*/*v*) ethanol and 50 µL of deionized water, two subsequent steps of vortex were applied to each samples’ suspensions posed in vials with metal cylinders. Five technical replicates were applied onto the 96-spot silicon microtiter plate, and spots were allowed to air-dry. Spectra reproducibility of the replicates measurements was verified through label coherence and cluster purity criteria; afterwards, an average spectrum was generated by the replicates’ spectra of each sample passing the quality criteria using the OPUS 8.2.28 software. The spectral data obtained from the FTIR Biotyper were analyzed using linear discriminant analysis (LDA) and principal component analysis (PCA) to identify patterns and relationships among the isolates as previously described [9]. Similarities between the isolates were determined according to the average linkage method and correlation metric. Hierarchical cluster analysis was applied to group isolates to establish relatedness among samples. *C. parapsilosis* isolates were clustered according to the software-calculated cut-off value (0.967) and by the optimized cut-off value already established in our previous study, equivalent to 0.995 [9].

Regarding STR analysis, isolates were cultured on SDA plates at 37 °C for 48 h, followed by DNA extraction as described prior [16]. Next, two multiplex PCRs per isolate were performed using PCR reagents and thermal conditions as outlined earlier [17]. Subsequently, amplicons were diluted 200 times in water and 10 µL with 0.12 µL of Orange 600 DNA size standard (Nimagen, Nijmegen, The Netherlands). Diluted amplicons were analyzed on a 3500 XL genetic analyzer (Applied Biosytems, Foster City, CA, USA). Copy numbers were assigned with GeneMapper 5 software (Applied Biosystems), and the phylogeny was inferred as described earlier [17]. Briefly, stutter peaks below 50% of the intensity of the highest peak for an allele, minus-A peaks and blead-through peaks were discarded. The resulting size for the STR allele was rounded. Relatedness between isolates was analyzed using BioNumerics software v7.6.1 (Applied Maths NV, Sint-Martens-Latem, Belgium) via the unweighted pair group method with arithmetic means (UPGMA), using the multi-state categorical similarity coefficient.

## 3. Results

### 3.1. Antifungal Susceptibility Testing

The results for the nine antifungal susceptibility profiles relative to the *C. parapsilosis* resistant isolates and surveillance positive swabs included in the study are summarized in Table S1.

According to the interpretive CLSI breakpoints, all isolates showed susceptibility to anidulafungin (AND), caspofungin (CAS), and micafungin (MF), with MICs ranging from 0.25 to 1 µg/mL. Isavuconazole (ISA) and posaconazole (PZ) exhibited very low MIC values across all isolates, indicating consistent susceptibility. Voriconazole (VOR) susceptibility varied, with most isolates showing MICs of 1 to 4 µg/mL. Itraconazole (ITZ) susceptibility was high, with MICs mostly at 0.06 to 0.25 µg/mL. Fluconazole (FLZ) resistance was notable, with MIC values reaching up to 256 µg/mL for the majority of the isolates. All other isolates had MICs of 128 µg/mL, indicating significant resistance levels. Lastly, amphotericin B (AB) showed consistent low MICs across all isolates, with values ranging from 1 to 2 µg/mL, indicating good susceptibility to this antifungal agent.

### 3.2. Sequencing Analysis

Sequencing analysis of the 26 isolates (24 clinical and two environmental) from the CRT facility identified single nucleotide polymorphisms (SNPs) conferring fluconazole resistance in *C. parapsilosis* [18]. The results revealed the presence of two key mutations in *ERG11*, Y132F and R398I, in various combinations across the isolates and a synonymous substitution (I197I). In particular, six isolates harbored both nonsynonymous mutations, the remaining 18 the *ERG^Y132F^* substitution only, and one case (CP-AR-231) harbored the R398I mutation only. A single wild-type susceptible isolate was added to the analysis (CP-AR-14) as an outgroup. The detailed mutation profiles of the clinical and surveillance isolates are summarized in Table S2.

Genetic similarities and differences are visible in the phylogenetic tree reported in Figure 1, clustering the isolates into distinct groups according to *ERG11* gene SNPs.

### 3.3. FTIR

**Figure 2:**
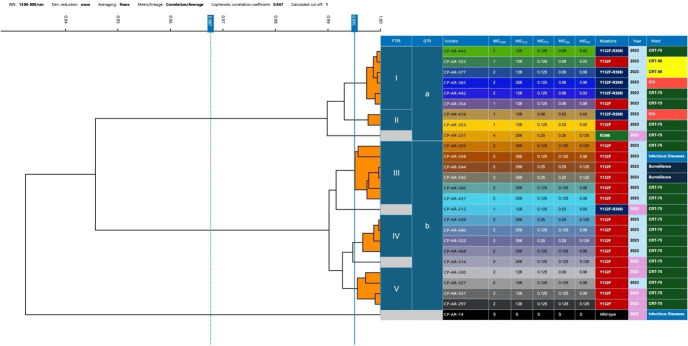
Dendrogram of *Candida parapsilosis* isolates clustered by FTIR spectral fingerprints.

The results of the Fourier-transform infrared (FTIR) spectroscopy analysis, as displayed in the dendrogram (Figure 2), allowed the *C. parapsilosis* isolates to cluster based on their spectral fingerprints.

At first, using the software calculated cut-off value of 0.967, based on their spectral data, it was possible to divide isolates into two big clusters indicating distinct groupings (a and b in Figure 2). Cluster “a” encompassed the majority of isolates harboring the *ERG11^Y132F-R398I^* substitution (five isolates). On the contrary, cluster “b” included one single isolate (CP-AR-212) with the *ERG11^Y132F-R398I^* substitution, while all the remaining isolates harbored the *ERG11^Y132F^* substitution only. Susceptible *C. parapsilosis* isolate CP-AR-14 clustered separately from all the rest of the samples. Interestingly, by applying the *C. parapsilosis* pre-established cut-off of 0.995 [9], an additional grouping of isolates was obtained. These additional clusters correlated with the detected mutations, reflecting the different substitutions along with the different outbreak isolate appearance time-line. Interestingly, the majority of isolates collected in the year 2022 clustered together in group V, with the exception of CP-AR-327 being isolated on the first day of 2023. Moreover, the set cut-off value allowed to distinguish isolate CP-AR-231, the only one harboring solely substitution *ERG11^R398I^,* as well as the *C. parapsilosis* susceptible isolate CP-AR-14 and isolates CP-AR-212 and CP-AR-214 as singletons according to their time of collection, belonging both to the first wave of spread that occurred during the year 2022. Notably, CP-AR-542 and CP-AR-544 isolates, retrieved from the 42 surveillance swabs, harbored the *ERG11^Y132F^* substitution and belonged to the same cluster III or b according to FTIR and STR analysis, respectively. In this branch were assigned four clinical isolates collected in the same year 2023, also harboring the *ERG11^Y132F^* substitution.

### 3.4. STR Genotyping

**Figure 3:**
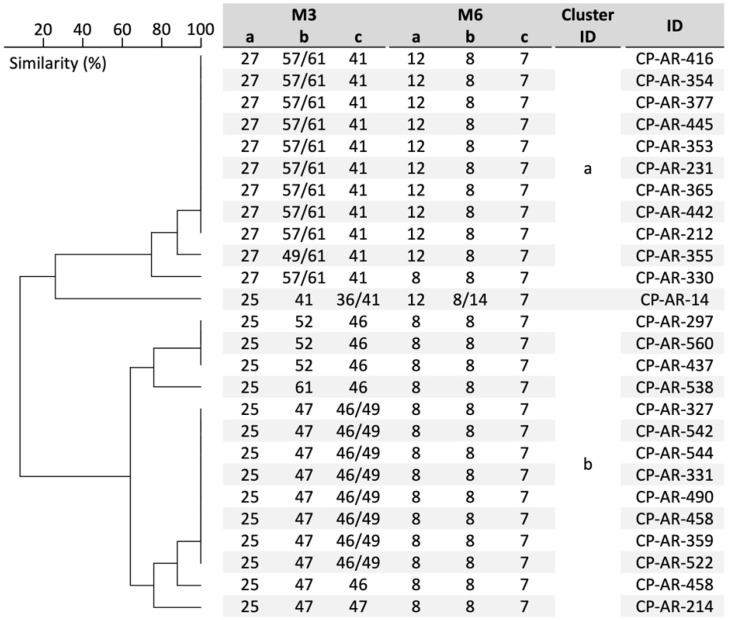
Cluster analysis based on short tandem repeat genotyping of 26 *Candida parapsilosis* isolates. The UPGMA dendrogram was generated with BioNumerics v7.5.

By amplifying six microsatellite alleles in all 26 *C. parapsilosis* isolates, nine genotypes were inferred, consisting of one to nine isolates (Figure 3). Three clusters were found that were made up of three to nine isolates and all had one or two highly related genotypes that only differed in a single marker, which were annotated with cluster ID a and b. When excluding the clusters and related genotypes, only CP-AR-14 was not related to other isolates, forming a singleton. When results were compared to FTIR genotyping, isolates were often allocated to the same cluster, except for isolates CP-AR-212 and CP-AR-330, coming to an agreement of 92%. With both methods, CP-AR-14 formed a singleton. Clusters I–V, as found by FTIR, were not discriminated with STR analysis.

## 4. Discussion and Conclusions

The spread of azole-resistant *C. parapsilosis* is strongly associated with hospital outbreaks of invasive infections, specifically centered on strains harboring the Y132F substitution in the *ERG11* gene, as reported in several studies in the last decade [5,18,19,20]. Contamination of medical devices and hospital surfaces can contribute to the persistence of this yeast in the environment and to its diffusion through healthcare operators, easily transmitted to the patients. Moreover, azole-resistant *C. parapsilosis* isolates seem to better adapt to the host’s conditions and survive longer in comparison to the susceptible strains [4]. As frequently reported in recent years [7,21], the emergence of such *C. parapsilosis ERG^Y132F^* mutant clones can contribute to a severe impact on mortality rates associated with candidemia in nosocomial settings.

Here, we discover the presence of the same mutations in the *ERG11* gene harbored by isolates from blood cultures and nosocomial surfaces, thus suggesting horizontal transmission of *C. parapsilosis* isolates. With both FTIR and STR genotyping, two large clusters were found (clusters I–II and III–V matching with STR clusters a and b, respectively), strengthening the indication for nosocomial transmission by two genetically distinct strains mostly dominated by different *ERG11* mutations. Nonetheless, whole genome sequencing (WGS) analysis is required to confirm clonal transmission. Previously, FTIR and STR results for *C. parapsilosis* were compared, although the agreement between the two methods was found to be lower, at 74%, while this was currently found to be 92% [9]. Nonetheless, the previous evaluation of the method referred to samples recovered from hospital-acquired BSI over an eight-year period, in contrast to the present study, which was conducted during the outbreak wave. This difference might explain the higher percentage of agreement reached and push further the application of the FTIR technique in a real-time scenario. While both methods are considered to have a high discriminatory power [22], the resolution of the STR scheme could further be improved by the inclusion of variable markers. Especially for the M6 panel, a low variety of alleles was found. Recently, a combination of markers from the M3 panel and others were proposed as the new STR scheme for *C. parapsilosis,* given the higher discriminatory power when compared to M6 markers [18]. Future investigation should genotype the current isolates with the newly proposed markers to confirm the higher discriminatory power.

Taken the genotyping results together, there are strong indications for nosocomial transmission, highlighting the need to keep attention on a systematic surveillance of wards that treat immunocompromised or at-risk patients, as demonstrated by the discovery of the two out of 42 surveillance swabs positive for *C. parapsilosis* fluconazole resistant strains.

Until now, outbreak surveillance strategies have been designed for bacterial and viral pathogens. *Candida auris* is the only yeast included in the existing platforms for genomic surveillance; nonetheless, not any approach is available or suggested for other *Candida* species, including *C. parapsilosis*.

Traditional identification methods for fungi often fall short in terms of speed and resolution, necessitating advanced techniques for better outbreak management. Of note, whole-genome sequencing (WGS) is the preferred method for determining genetic relationships among bacteria and viruses; however, its adoption for fungal surveillance faces challenges, including the larger size of fungal genomes, issues with ploidy, and variability in the genome as duplication and recombination. Once data are available, analysis is time consuming due to a lack of standardized bioinformatics tools and insufficient comparative genome databases [12]. These limitations imply that the use of this powerful technique for fungal outbreak tracking is not part of routine outbreak management. On the other end, the integration of FTIR spectroscopy and genetic sequencing by STR offers a robust approach for identifying and tracking fluconazole-resistant *C. parapsilosis* isolates during an outbreak.

One possible limitation to the applicability of FTIR methodology is that clustering cut-off value determination has not yet been determined for all the *Candida* species involved in clinically relevant outbreaks; further studies will be necessary to cover this pitfall in order to perform the IR Biotyper-based method on a wider number of emerging multidrug-resistant yeasts responsible for invasive candidiasis. Specifically, for *C. parapsilosis*, we were able to use the cut-off value set in our previous study [9]. Anyway, this is actually possible only for this *Candida* species and for *C. auris* [23] and *C. tropicalis* [24].

Moreover, a limitation of this study is that we did not perform WGS analysis on the clinical samples and positive environmental surveillance swabs collected during the fluconazole-resistant *Candida parapsilosis* outbreak at the rehabilitation clinic. However, although this analysis might have been useful to discover more clonal lineages, we were able to find the source of infection (monitor and over-bed table) and track the outbreak by FTIR, further confirming the isolates’ clustering by STR analysis.

In conclusion, this methodology enhances outbreak management by enabling rapid, precise identification and providing critical insights into the genetic diversity of the pathogen. The findings underscore the importance of advanced molecular techniques in effective infection control and highlight the potential for FTIR technology to improve and refine microbial identification in clinical microbiology. Moving forward, the continuous application of such advanced methods will be essential in managing and mitigating the impact of drug-resistant fungal pathogens with healthcare settings in the aim of providing timelier identification of *Candida* disease outbreaks. Therefore, we think that the FTIR spectroscopy approach will provide in the future for reliable real-time outbreak surveillance, thus making reality the unmet promise to predict nosocomial fungal clusters.

## Data Availability

The original data presented in the study are included in the article, further inquiries are available upon request to the corresponding authors.

## Supplementary Materials

The following supporting information can be downloaded at: https://www.mdpi.com/article/10.3390/jof10110753/s1, Table S1: Antifungal susceptibility tests. Table S2: *ERG11* gene mutations.
