# Supplementary material for: Follow the Path: Unveiling an Azole Resistant Candida parapsilosis Outbreak by FTIR Spectroscopy and STR Analysis
Source: J Fungi (Basel). 2024 Oct 30;10(11):753. doi: 10.3390/jof10110753 (PMC11595275; doi:10.3390/jof10110753)
Supplement: Supplementary file 1 [file jof-10-00753-s001.zip › Table S1rev.pdf]

TABLE S1. Antifungal susceptibility test

Drugs	MIC (µg/mL)														Range (µg/mL)	MIC 50	MIC 90	GM[a]	Mode[b]
	**256**	**128**	**64**	**32**	**16**	**8**	**4**	**2**	**1**	**0.5**	**0.25**	**0.125**	**0.06**	**0.03**					
**AND**	-	-	-	-	-	-	-	-	2	16	7	-	-	-	0.25-1	0.5	0.5	0.44	0.5
**MF**	-	-	-	-	-	-	-	2	17	6	-	-	-	-	0.5-2	1	1	0.90	1
**CAS**	-	-	-	-	-	-	-	-	9	15	1	-	-	-	0.25-1	0.5	1	0.62	0.5
**ISA**	-	-	-	-	-	-	-	-	-	-	5	10	7	3	0.03-0.25	0.125	0.125	0.09	0.125
**PZ**	-	-	-	-	-	-	-	-	-	-	-	8	11	6	0.03-0.125	0.06	0.125	0.06	0.06
**VOR**	-	-	-	-	-	-	1	19	5	-	-	-	-	-	1-4	2	2	1.79	2

**IZ**	-	-	-	-	-	-	-	-	-	-	5	19	1	-	0.06-0.25	0.125	0.125	0.13	0.125
**FLZ**	13	12	-	-	-	-	-	-	-	-	-	-	-	-	128-256	256	256	183.55	256
**AB**	-	-	-	-	-	-	-	2	23	-	-	-	-	-	1-2	1	1	1.06	1

Anidulafungin (AND), micafungin (MF), caspofungin (CAS), isavuconazole (ISA), posaconazole (PZ), voriconazole (VOR), itraconazole (ITZ), fluconazole (FLZ), and amphotericin B (AB). [a] Geometric mean calculated by multiplying all the MIC values and taking the n-th root, being n the number of values in the dataset [b] Mode calculated as the most frequently appeared MIC value in the dataset.
