# Supplementary material for: Follow the Path: Unveiling an Azole Resistant Candida parapsilosis Outbreak by FTIR Spectroscopy and STR Analysis
Source: J Fungi (Basel). 2024 Oct 30;10(11):753. doi: 10.3390/jof10110753 (PMC11595275; doi:10.3390/jof10110753)
Supplement: Supplementary file 1 [file jof-10-00753-s001.zip › Table S2rev.pdf]

Table S2. *ERG11* gene mutations. The table summarizes the *ERG11* Jane mutations Y132F, I197I and R398I along with their corresponding substitutions.

C. parapsilosis isolates	Month-year[a]	Y132F	I197I	R398I	Substitutions
CP-AR-14	Feb-2022	A	T	G	*wild-type*
CP-AR-212	Aug-2022	T	C	T	Y132F-R398I
CP-AR-214	Aug-2022	T	C	G	Y132F
CP-AR-231	Aug-2022	A	C	T	R398I
CP-AR-297	Nov-2022	T	C	G	Y132F
CP-AR-327	Jan-2023	T	C	G	Y132F
CP-AR-330	Dec-2022	T	C	G	Y132F
CP-AR-331	Dec-2022	T	C	G	Y132F
CP-AR-353	Feb-2023	T	C	G	Y132F
CP-AR-354	Feb-2023	T	C	G	Y132F
CP-AR-355	Feb-2023	T	C	G	Y132F
CP-AR-358	Feb-2023	T	C	G	Y132F
CP-AR-359	Feb-2023	T	C	G	Y132F
CP-AR-365	Feb-2023	T	C	T	Y132F-R398I
CP-AR-377	Mar-2023	T	C	T	Y132F-R398I
CP-AR-416	Apr-2023	T	C	T	Y132F-R398I
CP-AR-437	May-2023	T	C	G	Y132F
CP-AR-442	May-2023	T	C	T	Y132F-R398I
CP-AR-445	Jun-2023	T	C	T	Y132F-R398I
CP-AR-458	Jun-2023	T	C	G	Y132F
CP-AR-490	Aug-2023	T	C	G	Y132F
CP-AR-522	Sep-2023	T	C	G	Y132F
CP-AR-538	Nov-2023	T	C	G	Y132F
CP-AR-542[b]	Nov-2023	T	C	G	Y132F
CP-AR-544[b]	Nov-2023	T	C	G	Y132F
CP-AR-560	Dec-2023	T	C	G	Y132F

[a]Month and year of samples collection. [b] Environmental swabs samples from monitor (CP-AR-542) and over-bed table (CP-AR-544).
